# Construct validity of the Spanish version of the Post-COVID-19 Functional Status scale and validation of the web-based form in COVID-19 survivors

**DOI:** 10.1371/journal.pone.0269274

**Published:** 2022-06-01

**Authors:** Cristina Sacristán-Galisteo, Tamara del Corral, Marta Ríos-León, Patricia Martín-Casas, Gustavo Plaza-Manzano, Ibai López-de-Uralde-Villanueva

**Affiliations:** 1 Centro de Salud Las Fronteras, Gerencia Asistencial de Atención Primaria, Madrid, Spain; 2 Department of Radiology, Rehabilitation and Physiotherapy, Faculty of Nursing, Physiotherapy and Podiatry, Complutense University of Madrid, IdISSC, Madrid, Spain; 3 SESCAM, Toledo, Spain; Seoul National University College of Medicine, REPUBLIC OF KOREA

## Abstract

**Objectives:**

To assess the psychometric characteristics of the Spanish Post-COVID-19 Functional Status (PCFS) scale (web-based and paper-based forms) and the test-retest reliability of the web-based form.

**Study design and setting:**

Cross-sectional study of 125 COVID-19 survivors. The test-retest reliability of the web-based form was assessed at 7 days after the first evaluation. We collected symptoms, functional status (PCFS scale), health-related quality of life (EuroQol-5D questionnaire, EQ-5D-5L), activities of daily living limitations [Barthel Index and Global Activity Limitation Index, GALI] and psychological state (Hospital Anxiety and Depression Scale, HADS).

**Results:**

The paper- and web-based forms of the Spanish PCFS scale showed adequate construct validity, and the web-based form provided substantial test-retest reliability (kappa = 0.63). The percentage of agreement between the web-based and paper-based forms was high (88%). Functional status showed a high correlation with EQ-5D-5L (inverse) and GALI (direct) (both; Rho ≥ .743), a moderate correlation with HADS (Rho ≥ .409) and a low correlation with the Barthel Index (Rho < .30). The Kruskal–Wallis test showed statistically significant differences in EQ-5D-5L, GALI and HADS according to the degree of functional status.

**Conclusion:**

The Spanish version of the PCFS scale (web-based and paper-based forms) showed adequate construct validity, and the web-based form provided substantial test-retest reliability in COVID-19 survivors.

## Introduction

On March 11, 2020, the World Health Organization declared the outbreak of a global pandemic of novel severe acute respiratory syndrome coronavirus type 2 (SARS-CoV-2) causing coronavirus disease 2019 (COVID-19). Substantial numbers of SARS-CoV-2-convalescent patients experience symptoms months after acute infection and have not returned to their initial health state prior to infection, raising the suspicion of post-COVID-19 syndrome [1, 2]. These long-term health consequences have a significant impact on the physical, cognitive, mental and social health status, even in patients with mild disease presentation [3–5], suggesting a long-term functional decline in COVID-19 survivors.

The continued assessment of patients with post-COVID-19 syndrome has become a major task, with the aim of defining and mitigating the socioeconomic and medical long-term effects of COVID-19. In the absence of a specific instrument to assess the degree of impairment in the functional status of individuals who have had COVID-19, various physical and psychological dimensions have been initially examined. Among the tests used for this purpose were the 6-minute walk test, the 1-minute sit-to-stand test and the Barthel Index, as well as scales used to measure dyspnoea, anxiety, depression and quality of life [6–10]. An optimal instrument is needed to assess the functional status/degree of disability post-COVID-19 and to determine the impact of the disease on daily activities and lifestyle changes. For this reason, Klok et al. [11] developed the Post-COVID-19 Functional Status (PCFS) scale, an ordinal 6-grade scale ranging from 0 (“no functional limitations”) to 5 (“death”), which is used as a patient-reported outcome measure to evaluate the consequences of COVID-19 on functional status. The PCFS scale has been validated for Dutch and Belgian populations, showing adequate construct validity [12]. The linguistic validation and cross-cultural adaptation of the Spanish version was performed previously in the Chilean population, showing conceptual and linguistic equivalence to the original instrument [13]; however, a construct validity analysis has not been performed.

The COVID-19 pandemic has forced healthcare providers to be adaptive and innovative in their patient care, to maintain adequate social distancing and to reduce the risk of disease transmission. Among the measures taken, hospital visits have been limited to a minimum, and telematic consultations have increased, becoming alternatives to conventional appointments [14]. It is therefore relevant to evaluate the metric properties of a self-administered web-based form of the Spanish version of the PCFS scale to assess functional status and to monitor a patient’s progression, without requiring their attendance.

The aim of this study was to assess the psychometric characteristics of the Spanish version of the PCFS scale (web-based and paper-based forms) and evaluate the test-retest reliability of the web-based form.

## Materials and methods

### Study design, settings and participants

This cross-sectional survey study was conducted in Madrid (Spain) between February 16 and April 16, 2021. The study was approved by the local ethics committee of Clínico San Carlos Hospital (Madrid) (21/039-E) and was conducted in accordance with the Declaration of Helsinki. The fundamental rights of the participants were guaranteed, ensuring their well-being at all times, as well as being fully informed of their rights (e.g., the right to self-determination and the right to make informed decisions about their participation in the study, both at the beginning and during the course of the study). Written informed consent was obtained from all the participants. The sample was recruited from 2 healthcare centres in the Community of Madrid (Spain) area using a consecutive sampling by convenience method. The sample size was established based on the criteria of several authors, who have proposed 100 observations as the minimum required, as well as according to the proposed rule of a ratio of 20 observations per measured variable [15–17]. Thus, a necessary minimum of at least 100 participants were determined to analyze the construct validity of the scale. To capture the real-life scenario of COVID-19 survivors, the only selection criteria were as follows: confirmed SARS-CoV-2 infection (positive reverse-transcription–polymerase chain reaction [RT-PCR] test from a nasopharyngeal or oropharyngeal swab or serological tests positive for SARS-CoV-2 antibodies), persistence of symptoms for at least 30 days from the date of symptom onset, at least 18 years of age, able to speak Spanish and having internet access at home. Participants with pre-existing disability were excluded from the study. Written informed consent was obtained from all participants.

### Study procedures

The first evaluation required the attendance of the participants at the centre where the research was carried out. All the self-reported questionnaires in paper-based form were fulfilled during the interview (face-to-face), whereas the PCFS scale in web-based form was completed online. All participants answered both the paper- and web-based forms of the Spanish version of the PCFS scale on the same day with a minimum of 2 hours and with distracting activities between the administration of the 2 scales. The web-based form of the PCFS scale was an exact copy of the paper version and was placed on a secured Internet page. Participants could answer the questions by clicking the appropriate box; after finishing the scale, the results were submitted. The rest of the self-reported questionnaires were randomised and were completed during the interview. To assess the test-retest reliability of the web-based form, an invitation to complete the scale a second time was sent to the participants by e-mail 7 days after the first evaluation.

### Outcomes

A physician collected data by interviewing the participants, including sociodemographic characteristics (sex, age, height, weight, educational level, occupation, marital and smoking status, date of symptoms onset and type of COVID-19 diagnosis), comorbidities and clinical characteristics (dyspnoea, fatigue/muscle weakness).

#### Functional status

The consequences of COVID-19 on functional status were evaluated with the PCFS [11], an ordinal 6-grade scale: grade 0 (no functional limitations); grade 1 (negligible functional limitations); grade 2 (slight functional limitations); grade 3 (moderate functional limitations); grade 4 (severe functional limitations); and grade 5 (death). The scale was applied through a structured interview by a physician previously trained to validate the paper-based form and was self-administered by the participants to validate the web-based form. The final rating on the scale is the poorest functional status indicated by the participants’ answers.

#### Health-related quality of life

To assess the participant’s quality of life, we employed the European Quality of Life-5 Dimensions questionnaire (EQ-5D-5L) [18], which consists of 5 dimensions (1, mobility; 2, self-care; 3, usual activities; 4, pain/discomfort; 5, anxiety/depression) with 5 response options based on severity level, ranging from 1 to 5. Based on these 5-dimension codes, an index score is provided, ranging from 0 (death) to 1 (full health). Additionally, the participants had to rate their current overall health on a visual analogue scale ranging from 0 (worst imaginable health) to 100 (best imaginable health).

#### Activities of daily living limitations

Limitations in activities of daily living were evaluated by the Barthel Index [19] and Global Activity Limitation Index (GALI) [20]. The Barthel Index is an ordinal scale comprising 10 activities of daily living (feeding, grooming, bathing, dressing, bowel and bladder care, toilet use, ambulation, transfers and stair climbing) and is scored in steps of 5 points, resulting in a maximum total score of 100, with higher scores indicating more independence. The GALI is self-reported and asks *“For the past 6 months at least*, *to what extent have you been limited because of a health problem in activities people usually do*?*”*, to which there are 3 possible responses: not limited at all, limited but not severely and severely limited.

#### Anxiety and depression levels

The Spanish version of the Hospital Anxiety and Depression Scale (HADS) [21], which consists of 14 items divided into 2 subscales for anxiety and depression, was used to assess the patient’s level of anxiety and depression. The subscales include 7 items each, with scores ranging from 0 to 21 for each item.

### Data analysis

The data were analysed using SPSS v.25 software (SPSS Inc., Chicago, IL, USA). The significance level was set at P < 0.05. Continuous variables are presented as mean ± standard deviation or median and interquartile range, as appropriate. Categorical variables are presented as absolute and relative frequencies.

The percent agreement and kappa coefficients [22] were employed to estimate the concordance between the web-based and paper-based forms of the Spanish version of the PCFS scale, as well as the test-retest concordance after 7 days for the web-based form. Kappa values were interpreted according to Landis and Koch’s criteria [23]: poor agreement (<0.2), fair agreement (0.21–0.40), moderate agreement (0.41–0.60), substantial agreement (0.61–0.80), and almost perfect agreement (>0.80).

Construct validity was assessed by analysing the correlations between the scores of both PCFS scale forms with those obtained for quality of life (EQ-5D-5L), limitations in activities of daily living (GALI and Barthel Index) and psychological status (HADS). Spearman’s Rho correlation coefficient was used to determine the correlations due to the categorical nature of certain variables and the fact that the continuous variables did not have a normal distribution. A low correlation was considered <0.30, a moderate correlation 0.30–0.60, and a strong correlation >0.60 [24]. In addition, the discriminant ability of the Spanish version of the PCFS scale was assessed by comparing the scores obtained for quality of life, limitations of activities of daily living and psychological status between the various functional status grades. The functional status factor was analysed by employing the Kruskal–Wallis test, and multiple comparisons were performed with the Mann–Whitney U test.

## Results

The total sample consisted of 125 participants (71 women and 54 men; 46.46 ± 14.18 years; 25.50 ± 4.5 kg/m^2^) who had a confirmed diagnosis of COVID-19 by RT-PCR. In addition, 16 (12.8%) participants required hospitalisation during the COVID-19 infection (5 of whom required Intensive Care Unit admission), and only 5 (4%) had a comorbidity. Most participants had a grade 3 functional status according to the PCFS scale, regardless of the form used (web-based form, 34.4%; paper-based form, 35.2%). **Table 1** shows the participants’ sociodemographic and clinical characteristics. It is important to note that 4 participants did not complete the web-based form of the Spanish PCFS scale within 7 days of the first assessment. Hence, the test-retest agreement was analysed with a sample of 121 participants.

**Table 1:** Sociodemographic and clinical characteristics of the participants.

Outcomes	N (%)	Mean ± SD	Median (IQR)
**Age** (years)		46.46 ± 14.18	45 (36–57)
**Body Mass Index** (kg/m^2^)		25.50 ± 4.5	24.77 (22.40–27.44)
Underweight	3 (2.4%)		
Normal weight	63 (50.4%)		
Overweight	41 (32.8%)		
Obesity	18 (14.4%)		
**Level of education**			
School graduate	38 (30.4%)		
Intermediate training cycle	21 (16.8%)		
Higher level training cycle	9 (7.2%)		
University degree	57 (45.6%)		
**Occupation**			
Student	7 (5.6%)		
Active worker	74 (59.2%)		
Unemployed	14 (11.2%)		
Retired	14 (11.2%)		
Temporary incapacity/ sick leave	16 (12.8%)		
**Marital status**			
Alone	32 (25.6%)		
Married or living together	87 (69.6%)		
Divorced	4 (3.2%)		
Widow/er	2 (1.6%)		
**Smoking status**			
Never smoked	91 (72.8%)		
Current smoker	12 (9.6%)		
Ex-smoker	22 (17.6%)		
**Date of onset of symptoms** (days)		187.86 ± 133.03	137 (61.5–337)
**Severity of COVID-19**			
Severe (Intensive Care Unit admission)	5 (4%)		
Moderate (hospital admission)	11 (8.8%)		
Mild (no admission)	109 (87.2%)		
**Presence of Comorbidities**	5 (4%)		
**Presence of Dyspnoea**	78 (62.4%)		
**Presence of Fatigue/muscle weakness**	63 (50.4%)		
**Post-COVID-19 Functional Status Scale (web-based; paper-based forms)**			
Grade 0	36 (28.8%); 30 (24%)		
Grade 1	19 (15.2%); 28 (22.4%)		
Grade 2	20 (16%); 17 (13.6%)		
Grade 3	43 (34.4%); 44 (35.2)		
Grade 4	7 (5.6%); 6 (4.8%)		
**EuroQol 5D-5L**			
Index score (0–1)		0.799 ± 0.205	0.857 (0.680–1)
Visual Analogue Scale (0–100)		69.24 ± 19.47	70 (55–80)
**Global Activity Limitation Indicator**			
Not limited at all	60 (48%)		
Limited but not severely	53 (42.4%)		
Severely limited	12 (9.6%)		
**Barthel Index** (0–100)		99.68 ± 3.16	100 (100–100)
**Hospital Anxiety and Depression Scale**			
Anxiety (0–21)		7.14 ± 4.84	6 (3.5–11)
Depression (0–21)		4.61 ± 4.32	3 (1–7)
Total Score (0–42)		11.75 ± 8.61	9 (5–18)

### Agreement between the two forms and test-retest concordance for the web-based form

The percentage of agreement for the Spanish version of the PCFS scale rating between the web-based and paper-based forms was almost perfect when the scale was completed on the same day (both completions spaced approximately 2 hours apart), showing 88% agreement and a Kappa coefficient of 0.84 (0.76–0.92). Therefore, the two forms showed almost identical results and could apparently be applied interchangeably.

Regarding the stability of the score obtained again in the web-based form at 7 days (test-retest), the percentage of substantial agreement for the functional status grade was 72% (Kappa = 0.63 [0.52 to 0.74]). Thus, the stability over time of the web-based form of the PCFS scale was adequate.

### Construct validity

**Fig 1:**
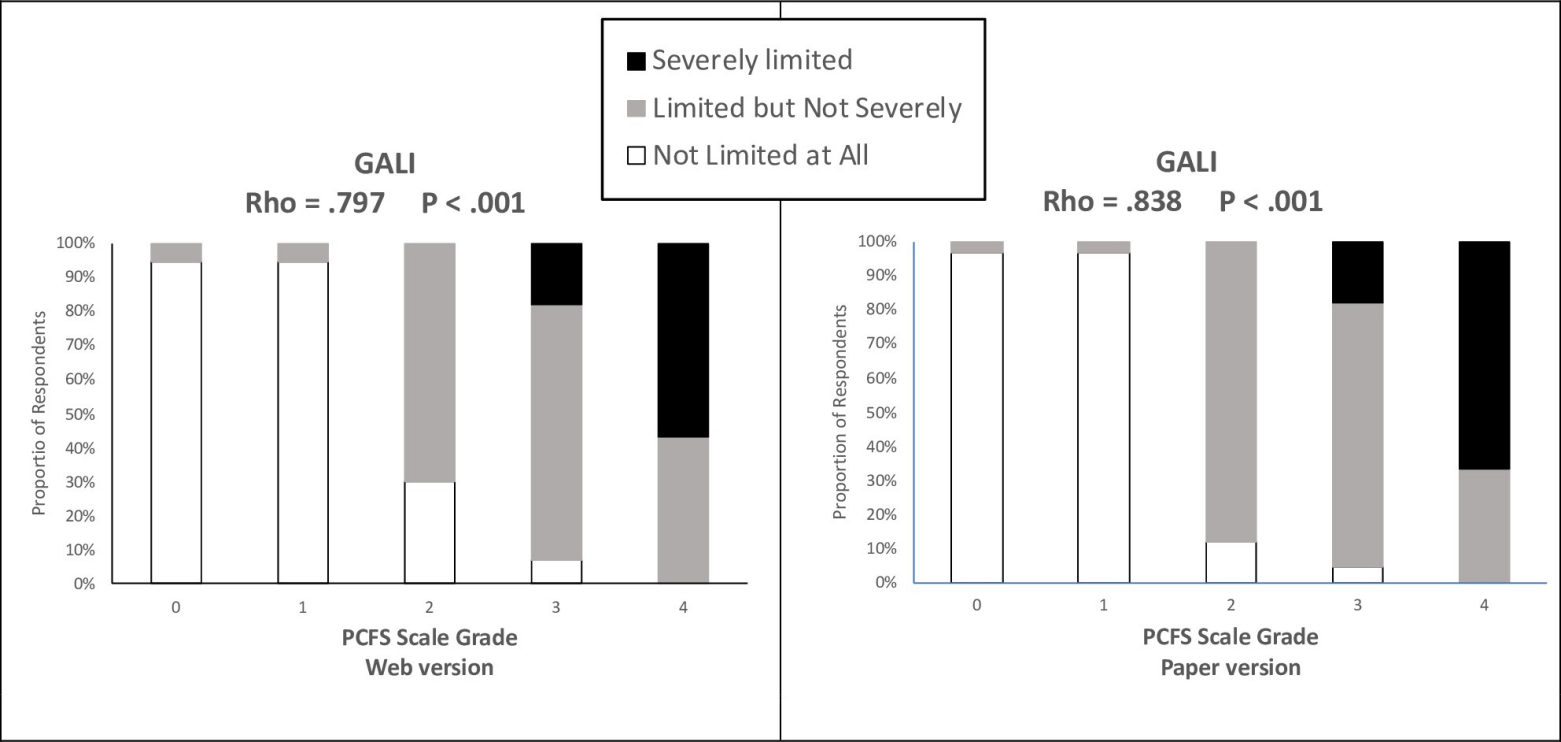
Associations between level of impairment in functional status and different degrees of the Global Activity Limitation Indicator (GALI). Abbreviatures: GALI, Global Activity Limitation Indicator; PCFS, Post-COVID-19 Functional Status.

The convergent validity of the PCFS scale was adequate, given that the correlational analysis showed a statistically significant relationship between the participant’s functional status and their quality of life, limitations in activities of daily living and psychological status (**Table 2**). Specifically, an increase in functional status was associated with an improvement in quality of life and psychological status, as well as less limitation in activities of daily living. All correlations between the Spanish PCFS scale, regardless of the form used, were of moderate-large magnitude, with the exception of those observed with the Barthel Index, which were low (Rho < .30). Functional status showed a particularly high correlation with quality of life (EQ-5D-5L; inverse correlation) and with limitation of usual activities of daily living (GALI; direct correlation) (**Fig 1**), with an absolute Rho value ≥ .743 in all cases.

**Table 2:** Spearman correlations between functional status and quality of life, limitations in activities of daily living and psychological status.

Outcomes	Post-COVID-19 Functional Status Scale	
	Web-based form	Paper-based form
**European Quality of Life-5 Dimensions (EQ-5D-5L)**		
Index score	-.831**	-.839**
Visual Analogue Scale	-.743**	-.752**
**Global Activity Limitation Indicator (GALI)**	.797**	.838**
**Barthel Index**	-.217*	-.218*
**Hospital Anxiety and Depression Scale (HADS)**		
Anxiety	.409**	.433**
Depression	.657**	.666**
Total Score	553**	566**

** P < 0.01

The Kruskal–Wallis test showed statistically significant differences in quality of life, limitation in activities of daily living and psychological status according to the functional status (P < .001). **Table 3** shows the multiple comparisons between participants with differing degrees of functional impairment according to the PCFS scale. Regardless of the form of the PCFS scale used (web-based or paper-based versions), statistically significant differences in the EQ-5D-5L and GALI were observed between almost all grades of functional status, except in the comparison between grades 0 and 1. These differences showed a deterioration in quality of life and an increase in limitations in activities of daily living as the degree of functional impairment increased. Only those participants classified as grade 4 on the PCFS scale showed a statistically significant deterioration with respect to the other participants, with lower functional status grades for performing of activities of daily living as assessed by the Barthel Index. In addition, the participants with a higher degree of functional impairment were more psychologically distressed, with statistically significant differences in the levels of anxiety and depression between functional status grades 3–4 and the other grades.

**Table 3:** Multiple comparisons between participants with different degrees of functional status impairment according to the PCFS scale for quality of life, limitations in activities of daily living and psychological status.

	Post-COVID-19 Functional Status Scale									
	Web-based form					Paper-based form				
	Mean ± SD					Mean ± SD				
	Median (first quartile–third quartile)					Median (first quartile–third quartile)				
	Grade 0 (N = 36; 29%)	Grade 1 (N = 19; 15%)	Grade 2 (N = 20; 16%)	Grade 3 (N = 43; 34%)	Grade 4 (N = 7; 6%)	Grade 0 (N = 30; 24%)	Grade 1 (N = 28; 22%)	Grade 2 (N = 17; 14%)	Grade 3 (N = 44; 35%)	Grade 4 (N = 6; 5%)
**EQ 5D-5L**										
** Index score**	0.980 ± 0.043	0.928 ± 0.121	0.814 ± 0.116	0.656 ± 0.157	0.389 ± 0.120	0.984 ± 0.039	0.936 ± 0.102	0.793 ± 0.104	0.649 ± 0.154	0.369 ± 0.116
	1	1	0.830	0.680	0.397	1	1	0.769	0.680	0.381
	(1–1)	(0.897–1)^a^	(0.733–0.910) ^a^^,^^b^	(0.516–0.749) ^a^^,^^b^^,^^c^	(0.293–0.495) ^a^^,^^b^^,^^c^^,^^d^	(1–1)	(0.910–1) ^a^	(0.736–0.849) ^a^^,^^b^	(0.510–0.735) ^a^^,^^b^^,^^c^	(0.266–0.481) ^a^^,^^b^^,^^c^^,^^d^
** VAS**	86.25 ± 13.28	78.33 ± 11.38	68.75 ± 10.24	55.81 ± 16.07	40.71 ± 7.32	87 ± 13.43	78.75 ± 11.36	68.82 ± 10.08	55.45 ± 15.62	38.33 ± 4.08
	90	80	70	60	40	90	80	70	57.5	40
	(80–98.75)	(70–90) ^a^	(60–75)^a^^,^^b^	(46.25–70)^a^^,^^b^^,^^c^	(40–40)^a^^,^^b^^,^^c^^,^^d^	(80–100)	(70–90) ^a^	(62.5–75) ^a^^,^^b^	(46.25–65) ^a^^,^^b^^,^^c^	(37.5–40) ^a^^,^^b^^,^^c^^,^^d^
**GALI**	0.06 ± 0.23	0.06 ± 0.24	0.70 ± 0.47	1.12 ± 0.50	1.57 ± 0.54	0.03 ± 0.18	0.04 ± 0.19	0.88 ± 0.33	1.14 ± 0.46	1.67 ± 0.52
	0	0	1	1	2	0	0	1	1	2
	(0–0)	(0–0)	(0–1) ^a^^,^^b^	(1–1)^a^^,^^b^^,^^c^	(1–2)^a^^,^^b^^,^^c^^,^^d^	(0–0)	(0–0)	(1–1) ^a^^,^^b^	(1–1) ^a^^,^^b^^,^^c^	(1–2) ^a^^,^^b^^,^^c^^,^^d^
**Barthel Index**	100 ± 0.0	100 ± 0.0	100 ± 0.0	100 ± 0.0	94.29 ± 13.05	100 ± 0.0	100 ± 0.0	100 ± 0.0	100 ± 0.0	93.33 ± 14.02
	100	100	100	100	100	100	100	100	100	100
	(100–100)	(100–100)	(100–100)	(100–100)	(95–100)^a^^,^^b^^,^^c^^,^^d^	(100–100)	(100–100)	(100–100)	(100–100)	(87.5–100) ^a^^,^^b^^,^^c^^,^^d^
**HADS**										
** Anxiety**	4.86 ± 3.65	6.33 ± 4.43	5.70 ± 3.61	9.79 ± 5.11	9.71 ± 4.68	4.43 ± 3.58	6.07 ± 4.12	6.06 ± 3.72	9.64 ± 5.13	10.50 ± 4.59
	4	5.5	5	11	11	4	5.5	5	10.5	11.5
	(2–6)	(2.75–9)	(3.25–9.25)	(4.25–14)^a^^,^^b^^,^^c^	(5–15)^a^^,^^c^	(2–6)	(3–9)	(3.5–10.5)	(5–14) ^a^^,^^b^^,^^c^	(5.5–15) ^a^^,^^b^^,^^c^
** Depression**	1.83 ± 2.43	2.89 ± 3.22	3.10 ± 2.51	7.79 ± 4.48	8.29 ± 3.09	1.53 ± 2.22	3.11 ± 2.94	2.76 ± 2.11	7.80 ± 4.48	8.83 ± 2.99
	1	2	2.5	6.5	8	1	2	2	7	9
	(0–2.75)	(1–4.25)	(1–4.75) ^a^	(4–11)^a^^,^^b^^,^^c^	(5–12)^a^^,^^b^^,^^c^	(0–2)	(1–4.75) ^a^	(1–4.5) ^a^	(4–11) ^a^^,^^b^^,^^c^	(5.75–12) ^a^^,^^b^^,^^c^
** Total score**	6.69 ± 5.59	9.22 ± 6.84	8.80 ± 5.63	17.58 ± 8.92	18 ± 7.62	5.97 ± 5.40	9.18 ± 6.12	8.82 ± 5.38	17.43 ± 9.01	19.33 ± 7.39
	5	7.5	8	17	19	5	8	8	17.5	21.5
	(3–8.75)	(4.75–10.75)	(5–12.25)	(8.25–24.75) ^a^^,^^b^^,^^c^	(10–25) ^a^^,^^b^^,^^c^	(3–8)	(5.25–11.5) ^a^	(4.5–14) ^a^	(9–24.75) ^a^^,^^b^^,^^c^	(11.25–25.5) ^a^^,^^b^^,^^c^

**Abbreviatures:** EQ 5D-5L, European Quality of Life-5 Dimensions; VAS, Visual Analogue Scale; GALI, Global Activity Limitation Indicator; HADS, Hospital Anxiety and Depression Scale.

^a^ significant differences with respect grade 0

^b^ significant differences with respect grade 1

^c^ significant differences with respect grade 2

^d^ significant differences with respect grade 3

## Discussion

The present study assessed the psychometric characteristics of the Spanish version of the PCFS scale (web-based and paper-based forms), and the test-retest reliability of the web-based form. The results show for the first time the psychometric characteristics of the Spanish version of the PCFS scale, for both the web-based and paper-based forms, and the agreement between these 2 forms. Both forms showed adequate construct validity, and the web-based form provided substantial test-retest reliability. Scores derived through the 2 forms of the scale showed a high level of agreement between them, which suggests there was minimal within-subject variance between the 2 modes of administration. Thus, the paper and electronic format of the Spanish version of the PCFS scale can be used interchangeably in clinical practice for determining the functional status after COVID-19 in the Spanish-speaking population.

The percentage of agreement for the PCFS scale rating between the web-based and paper-based forms was high (88%). Prior published studies have validated an electronic or web-based form of a paper-based questionnaire, showing uniform psychometric properties between administration modes (pain assessments [25, 26] and respiratory symptoms [27, 28]). The COVID-19 pandemic has led to an unprecedented situation in which there has been a major push to implement telematic consultations, emphasising the need for assessment tools such as this web-based form, which offers the possibility to assess patients’ functional status without needing outpatient visits. The use of electronic data capture for health assessment offers data management capabilities, improved adaptability, enhanced ergonomics and appeal [29], as well as reducing the number of spoiled responses without altering the results [30].

Test-retest analyses for the online administration of the Spanish version of the PCFS scale demonstrated that data gathered with this method are stable over time with no notable changes in functional status. There was high concordance between the responses to the 2 testing modes, with a slightly lower decrease in concordance for the first day, as has been reported in other tests, such as the 6-minute walk test [31]. This result means that a longer time span increased the risk of a change in functional status control; thus, the time factor can determine the progression of functional status. This outcome highlights the scale’s sensitivity in detecting changes over time, as can be observed in the discriminant validity results.

The construct validity of the Spanish version of the PCFS scale, for both the web-based and paper-based forms, was adequate and similar to the original version [12]. All correlations between the scale and other validated tools, regardless of the form used (web-based or paper-based), were of moderate-large magnitude. Notably, the strongest associations were with the EQ-5D-5L and GALI scores, which is in line with a previous study by Machado et al. [12], who showed strong correlations with the “usual activities” domain of the EQ‐5D-5L questionnaire. A key element in explaining these results is that the PCFS scale [11] evaluates the same dimensions of functional limitations, including changes in lifestyle, sports and social activities, which are included in GALI [20], as well as frequent symptoms after COVID-19, such as pain, fatigue, anxiety and depression, which can reduce quality of life [32]. In fact, these associations were to be expected due to the fact that the course of the infection is mild or asymptomatic in approximately 80%–90% of cases [33]. Therefore, the survivors will perceive earlier functional limitations in their usual activities, such as employment and social activities, making this scale the most specific and comprehensive assessment for this condition.

We also found a moderate correlation between the PCFS scale and the psychological status listed in the HADS, which could partially be explained by the fact that it has been reported that psychological status is associated with functional impairment in patients with COVID-19 [34]. Moreover, the PCFS scale specifically inquiries about psychological factors; however, there are many other factors that can affect the functional status that could explain this moderate correlation. In contrast, the Barthel Index showed a significant but low correlation. Only those participants classified as grade 4 on the PCFS scale showed limitations in performing activities of daily living assessed by the Barthel Index. In fact, only 1 participant showed a total score <100 for the Barthel Index. These results are in line with those of other studies that showed low Barthel indices related to poorer functional ability after COVID-19 [6, 35, 36]. However, the low correlation observed suggests that the PCFS scale covers the entire range of severity for the functional status, allowing a precise discrimination of the dimensions affected in these patients, whereas the Barthel Index only detects functional limitations in activities of daily living in severely impaired individuals (ceiling effect). This conclusion disagrees with Pizarro-Pennaroll et al. [37] who recommend the use of the Barthel Index for assessing activities of daily living in patients post-COVID-19. One reason that could explain these contradictory results was that our sample consisted of nonhospitalised COVID-19 survivors with mild disease presentation.

The discriminant ability of the Spanish version of the PCFS scale has been demonstrated, given that the participants classified by the scale with differing grades of functionality also showed differences in quality of life, performance of activities of daily living and psychological status. These results are similar to those reported for the original version [11, 12]. According to our findings, the discriminant validity was particularly reinforced by the differences observed between the various grades of functionality established by the PCFS scale and the quality of life and activities of daily living evaluated by the EQ-5D-5L and GALI, respectively. These results are supported by a recent review that suggested that the EQ-5D-5L is an optimal tool for detecting differences related to COVID-19 severity [37]. In addition, the distress grade was higher in those participants with grade ≥ 3 measured by the PCFS scale. A possible explanation is that severe limitations in performing activities of daily living, such as employment, leisure activities and/or personal hygiene, logically affect the individual’s emotional state.

The first limitation of the current study is the limited external validity of the Spanish version of the PCFS scale given that most of the participants were nonhospitalised COVID-19 survivors. However, this version might be a reliable representation of the current population of nonhospitalised COVID-19 survivors with moderate long-term functional limitations. Future studies are needed to investigate the distribution of the scale grades in selected cohorts of individuals, such as hospitalised or post-discharge patients with COVID-19, to demonstrate associations between functional status and response to therapy and linking functional status measures to patient preferences and utilities. Another limitation lies in the potential difficulties with web-based monitoring in less literate or technologically sophisticated populations, which warrants further research in these populations.

The Spanish version of the PCFS scale is a validated tool to assess the degree of impairment in functional status in the Spanish population who have had COVID-19, and to generate new therapeutic approaches in the rehabilitation programs, guide post-COVID-19 care, monitor the recovery process and assess functional sequelae, all of which could help in making treatment decisions on the multidisciplinary interventions aimed at improving the psychological state, which will presumably lead to an increase in quality of life. With an increasing number of primary care practitioners and specialists using electronic resources due to the COVID-19 pandemic, the need to have validated electronic forms of scales is becoming paramount. This electronic form of the scale provided results comparable to the paper form; thus, its utilisation can aid healthcare practitioners collecting and analysing the consequences of COVID-19 on functional status for long-term management without the need for outpatient visits.

## Conclusion

This study provides evidence of the psychometric characteristics of the Spanish version of the PCFS scale, both the web-based and paper-based forms, in COVID-19 survivors. Both versions showed adequate construct validity, and the web-based form provided substantial test-retest reliability. The comparability of a web-based form with the existing paper-based form allows the use of the web-based form in clinical practice. The Spanish version of the PCFS scale is an adequate tool for determining the degree of functional status after COVID-19 in the Spanish-speaking population.
